# Frequent Genital HSV-2 Shedding among Women during Labor in Soweto, South Africa

**DOI:** 10.1155/2014/258291

**Published:** 2014-05-20

**Authors:** Tara Perti, Mandisa Nyati, Glenda Gray, Guy De Bruyn, Stacy Selke, Amalia Magaret, Meei-Li Huang, Sithembiso Velaphi, Lawrence Corey, Anna Wald

**Affiliations:** ^1^Department of Medicine, University of Washington, Seattle, WA, USA; ^2^Perinatal HIV Research Unit, University of the Witwatersrand, Johannesburg, South Africa; ^3^Department of Laboratory Medicine, University of Washington, Seattle, WA, USA; ^4^Vaccine and Infectious Diseases Division, Fred Hutchinson Cancer Research Center, Seattle, WA, USA; ^5^Department of Pediatrics, Chris Hani Baragwanath Hospital, University of the Witwatersrand, Johannesburg, South Africa; ^6^Department of Epidemiology, University of Washington, Seattle, WA, USA

## Abstract

*Background*. Despite high herpes simplex virus type 2 (HSV-2) incidence and prevalence among women in Africa, we are unaware of published neonatal herpes reports. To assess neonatal HSV transmission potential in South Africa, we investigated the frequency of the strongest risk factors: HSV acquisition in late pregnancy and HSV shedding during labor. *Methods*. Women admitted in early labor to a hospital in Soweto underwent HSV serologic testing and genital swab collection for HSV PCR. HSV-2 seronegative women were assessed for seroconversion 4–6 weeks after delivery. *Results*. Of 390 women enrolled, 229 (58.7%) were HSV-2 seropositive. Genital HSV-2 was detected in 17.2% of HSV-2 seropositive women, including 26 of 115 HIV-positive and 13 of 110 HIV-negative women (22.6% versus 11.8%; RR, 1.91; 95% CI, 1.04–3.53; *P* = 0.038), but in none of 161 HSV-2 seronegative women. Among the 91 HSV-2 seronegative women followed after delivery, none seroconverted. *Conclusions*. HSV-2 reactivation is common among South African women during labor, especially those with HIV coinfection. To determine the epidemiology of neonatal herpes in South Africa and to investigate whether the lack of reported cases is due to alterations in immune control or HSV-2 virulence, studies evaluating acutely ill neonates for HSV and studies of maternal HSV-2 shedding patterns are needed.

## 1. Introduction

Neonatal herpes is one of the most deadly infections in the newborn. Untreated, mortality is 50% when the central nervous system (CNS) is involved and 85% for disseminated infection, with up to 50% of survivors demonstrating developmental abnormalities [1, 2]. Administration of high-dose acyclovir decreases mortality to 4% for CNS and 29% for disseminated disease [2]. Diagnosis of neonatal herpes, however, is challenging: vesicles are absent in nearly 40% of severe infections [2, 3]; other early symptoms and signs are nonspecific, and disseminated infection can appear identically to bacterial sepsis [4].

In the USA, estimates of neonatal herpes incidence range from 1/1,700 to 1/12,500 live births [4]. This risk is more than 300 times higher when herpes simplex virus (HSV) is isolated versus not isolated from the genital tract during labor [5]. More than half of neonatal herpes cases in the USA and Europe are associated with maternal acquisition of HSV-1 or HSV-2 near the time of delivery and the remainder result from exposure of the baby to reactivating maternal infection [5–7]. The rate of transmission to the infant is higher (25–50%) in first episode infections than reactivation (<1%), presumably due to the lack of transplacental transfer of maternal neutralizing antibodies and increased quantity of HSV shed in first episode infections [4, 5, 7, 8].

Routine serologic screening of pregnant women for HSV is not recommended in the United States [9]. Cesarean section is recommended for women with genital lesions or prodromal symptoms at delivery [9]. Anti-HSV therapy is recommended for women with a first episode of genital herpes during pregnancy to ameliorate their clinical symptoms. For women with recurrent genital herpes, suppressive therapy at 36 weeks of gestation is recommended to reduce the incidence of genital lesions and subsequent cesarean sections [9]. Routine HSV screening of pregnant women, HSV suppressive therapy near term, and cesarean section for women with genital lesions at delivery are not the standard of care for women with genital herpes in South Africa.

In South Africa, HSV-2 seroprevalence among women of reproductive age is considerably higher than in the USA (30–70% versus 21%) [10–13]. HSV-2 incidence in southern Africa remains high throughout the reproductive years, at 8.8/100 person-years among women 18–24 years of age and 5.3/100 person-years among women 35 years or older [14]. This is higher than the US, where estimated HSV-2 incidence is 2.25/100 person-years among women 20–29 years of age and 1.73/100 person-years among women age 30–39 [15] and where 1.0% of HSV-2 seronegative women seroconvert during pregnancy [7]. This suggests that South African women may also be at risk of HSV-2 infection during pregnancy. However, few case reports of neonatal herpes from developing countries and, to our knowledge, no reports from Africa have been published. This may reflect a lower incidence of neonatal herpes or underdiagnosis and underreporting, reflecting the nonspecific symptoms and signs of neonatal herpes, and the expense of laboratory testing for HSV in environments with a high burden of competing infectious diseases. To assess the potential for neonatal HSV transmission in South Africa, we performed a prospective cohort study investigating the frequency of HSV-2 acquisition in late pregnancy and HSV shedding from the genital tract of women during labor.

## 2. Materials and Methods

### 2.1. Study Population

We recruited women in early labor at the Chris Hani Baragwanath Maternity Hospital in Soweto, South Africa, including women undergoing induction. Entry criteria included women ≥18 years of age who were expected to have a vaginal delivery, known HIV status or plan for testing prior to delivery, ability to return to clinic in 4–6 weeks, ability to read and understand the consent form in English, Zulu, or Sotho, and willingness and ability to give written consent. The protocol was approved by the Institutional Review Boards of the University of the Witwatersrand and the University of Washington and all participants signed an informed consent.

### 2.2. Clinical and Laboratory Procedures

Baseline clinical data, including prenatal rapid plasma reagin (RPR) result, HIV status, and for, HIV-positive women, the most recent CD4 count and current antiretroviral drugs, were extracted from the participant's medical record. Participants were interviewed regarding history of genital ulcer disease and to confirm their antiretroviral regimen.

A swab of the genital skin and mucosa of the vagina, vulva, and perineum was collected with a polyester-tipped applicator [16, 17]. Participants were examined for genital lesions and a separate specimen was obtained from any lesions. Swabs were placed in a vial containing 1 mL of PCR digestion buffer and stored at room temperature. At the University of Washington, DNA was extracted from 200 *μ*L of buffer with a QIAamp 96 DNA Blood Kit (Qiagen) following the manufacturer's recommendations [18]. Quantitative real-time PCR was performed (QuantiTect multiplex PCR master mix from Qiagen) with a 7900HT sequencing detection system using a validated assay with common primers to the HSVgB region [18, 19]. A positive assay was defined as detection of ≥150 copies of HSV DNA/mL of swab fluid [19]. Positive samples were analyzed with type-specific primers to distinguish HSV-1 from HSV-2 [20].

Blood was drawn for HSV-2 serology. Kalon HSV-2 gG2 ELISA was performed in South Africa. Results were reported according to the manufacturer's specifications for index value cut-offs: >1.1 was positive, <0.9 was negative, and 0.9–1.1 was reported as equivocal. The serum was stored at −20°C and shipped to the University of Washington for confirmatory HSV Western blot [21].

Delivery data was extracted from participants' charts. At 10–14 days after delivery, participants were contacted by phone to assess the clinical status of the neonate. At the 4–6 week postpartum visit, the mother was interviewed about the interim medical history of the infant and the infant was examined. Women with negative or equivocal HSV-2 results by the Kalon assay at enrollment underwent repeat HSV Western blot to evaluate for seroconversion. If a participant did not return for the scheduled postpartum visit, attempts were made to contact the participant by phone; if unable to reach the participant, in those who had agreed to home visits, staff would visit the participant's home.

### 2.3. Definitions

HSV serostatus was defined by HSV-1 and HSV-2 antibody profiles obtained by Western blot [21]. Participants were classified into the following categories: HSV seronegative, HSV-1 seropositive only, HSV-2 seropositive only, and both HSV-1 and HSV-2 seropositive. HSV-2 seropositive women, therefore, included women only HSV-2 seropositive and women both HSV-1 and HSV-2 seropositive. Seroconversion was defined as a change in HSV status between acute (delivery) and convalescent (postpartum) sera. For participants with atypical Western blot profiles [22], acute and convalescent profiles were compared to assess for progressive acquisition of HSV bands. If no change was demonstrated, the results were considered negative. Among women with pre-existing HSV-1 antibodies who demonstrate HSV-2 seroconversion between labor and 4–6 weeks after delivery, most will have acquired HSV-2 in the last trimester of pregnancy, as 68% of persons with prior HSV-1 infection demonstrate HSV-2 seroconversion within 3 months after HSV-2 infection [22]. The followup time was selected to capture participants infected with HSV-2 in late pregnancy rather than in the postpartum period [22].

### 2.4. Statistical Analysis

The primary endpoint was the frequency of HSV acquisition in late pregnancy as determined by HSV shedding from the genital skin and mucosa of women lacking antibodies to the same HSV type, or seroconversion. Secondary endpoints were the frequency of genital HSV shedding in women with antibodies to the same HSV type and, among HSV-2 seropositive women, the risk of HSV-2 shedding for HIV-positive compared with HIV-negative women. Confidence intervals for proportions were obtained by the Agresti-Coull method [23]. When no events were observed, confidence intervals were determined by the mid-*P* method [24]. Poisson regression with robust variance estimates was used to determine the relative risk of genital HSV shedding for HIV-positive compared with HIV-negative women among those HSV-2 seropositive, and the relative risk of HSV-2 seropositivity for HIV-positive compared with HIV-negative women. The following covariates were tested for univariate association with genital HSV shedding and HSV-2 seropositivity: age, prior pregnancy, CD4 count, highly active antiretroviral therapy (HAART: defined as ≥three antiretroviral drugs), and HAART regimen containing tenofovir, an antiretroviral that inhibits HSV DNA polymerase [25]. Variables significant at *P* < 0.2 were included in a multivariate model. Backwards elimination was used to remove covariates not significantly (*P* < 0.05) associated with each outcome. The mean quantity of HSV DNA for positive samples was compared for HIV-positive and HIV-negative women by *t*-test.

Among HSV-2 seropositive women, Poisson regression was also used to determine the relative proportion of women reporting a history of genital ulcer disease for HIV-positive compared with HIV-negative women. The same covariates were tested for univariate association with history of genital ulcer disease and variables significant at *P* < 0.2 were included in a multivariate model followed by backwards elimination of nonsignificant (*P* < 0.05) variables. Analyses were performed using Stata 12.0 (StataCorp, College Station, TX).

Calculations of study precision estimated that between 239 and 474 participants would be required to estimate the proportion of women with recent HSV acquisition (in the past 3 months) with 1% precision, assuming a HSV-2 seroprevalence of 50% [10–12, 26] and an annual HSV-2 incidence between five to ten cases/100 person-years [14].

To assess whether HSV-2 seronegative participants for whom we did not obtain postpartum serum may have been more likely to seroconvert than those for whom we did obtain postpartum serum, we compared baseline risk factors in the two groups.

## 3. Results

We enrolled 390 women from whom we collected serum for HSV serology and genital swabs for HSV PCR. The median age of participants was 26 (range, 18–44) years; 135 (35.4%) were primigravid (Table 1). Of 387 women with known HIV status, 132 (34.1%) were HIV-positive; the median CD4 count (obtained at a median of 3 months prior to enrollment) was 321 (range, 18–1237) cells/*μ*L. All but one were receiving antiretroviral therapy, including 52 (39.7%) who were receiving HAART and 78 (59.5%) who were receiving antiretroviral drugs to prevent mother-to-child transmission. Five (1.3%) of 377 women had a positive RPR during the prenatal period, only one of whom reported a history of genital ulcers; this participant was also HSV-2 seropositive. No women were taking acyclovir.

### 3.1. Baseline HSV-2 Serostatus and History of Genital Ulcer Disease

**Figure 1:**
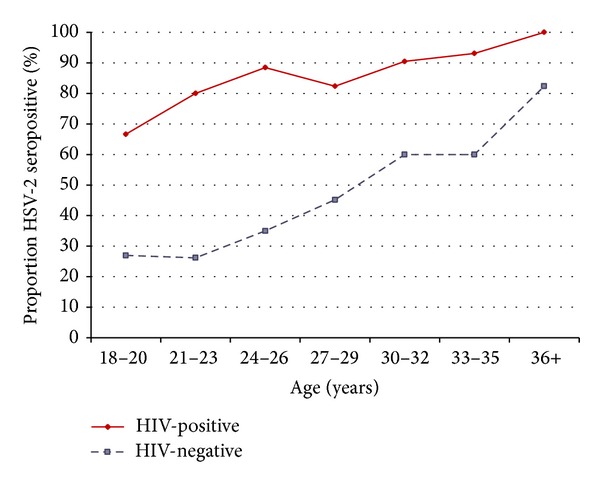
HSV-2 seroprevalence per age group, stratified by HIV status.

HSV-2 seropositivity, as determined by Western blot, was present in 229 women (58.7%), including 116 (87.9%) of 132 HIV-positive and 111 (43.5%) of 255 HIV-negative women (Table 2). HSV-2 seroprevalence increased with age (Figure 1). Of the 29 women who reported a history of genital ulcers, 22 (76%) were HSV-2 seropositive and reported a mean of four (range, 0–24) episodes in the past year compared to one episode (range, 0–2) for HSV-2 seronegative women. Reported genital ulcer disease was more common among HSV-2 seropositive women coinfected with HIV compared with HIV-negative women (14.2% versus 5.5%; relative risk, 2.57; 95% CI, 1.04–6.34; *P* = 0.040). Thirteen of 16 HIV-positive/HSV-2 seropositive women with a history of genital ulcer disease reported a mean of two episodes in the past year compared to nine episodes for six HIV-negative/HSV-2 seropositive women.

### 3.2. Genital HSV Shedding

Genital HSV shedding was detected in none of 161 HSV-2 seronegative women (95% CI, 0–1.8%). Among HSV-2 seropositive women, genital HSV was detected in 17.2% (39 of 227) and was typed as HSV-2 in all cases; all but two shedding episodes were subclinical. The risk of HSV shedding among HSV-2 seropositive women was higher for HIV-positive compared with HIV-negative women (22.6% versus 11.8%; relative risk, 1.91; 95% CI, 1.04–3.53; *P* = 0.038). The mean quantity of HSV-2 DNA was similar among HIV-positive and HIV-negative women (4.57 versus 4.42 log_10_ copies/mL; *P* = 0.80). Lesions were identified in three (2.6%) of 116 HSV-2/HIV-coinfected compared with four (3.6%) of 111 HSV-2 seropositive/HIV-negative women. HSV DNA was detected from only two women with lesions. Genital swabs were collected at a median of one day prior to delivery (range, 0–50 days). Subgroup analysis, including only those women for whom swabs were collected within one day of delivery, provided similar results: among HSV-2 seropositive women, HSV shedding was detected in 23% of 61 HIV-positive compared with 10% of 58 HIV-negative women.

### 3.3. HSV-2 Seroconversion

Postpartum serology was available for 91 (56.5%) of 161 HSV-2 seronegative women, collected at a median of 42 (range, 25–78) days after delivery. None seroconverted (95% CI, 0–3.2%). We found no significant differences in age, gravidity, parity, or HIV-status at baseline between HSV-2 seronegative participants who did and did not undergo postpartum HSV serology.

### 3.4. Neonatal Outcomes

Ten deaths occurred among infants during the follow-up period including five among liveborn infants within the first 28 days. The neonatal mortality rate of 5 per 394 live births (13/1000; 95% CI, 5–30/1000 live births) is similar to that estimated for South Africa (18-19/1000 live births) [28, 29]. None of the infants who died were evaluated for neonatal herpes. Three stillborn infants were delivered from HIV-negative women, two of whom were HSV-2 seropositive and none of whom had HSV-2 shedding detected (Table 3). Five neonates who died were also born to HIV-negative women, three of whom were HSV-2 seropositive and none of whom had HSV-2 shedding detected. Two infants died after the neonatal period, one at day 35 and one at day 36 of life. Both were born to women coinfected with HIV and HSV-2, and both women had genital HSV shedding detected. Including these two, 27 infants were identified as potentially exposed to HSV during delivery, as they were born to women with HSV shedding detected and were delivered vaginally or by cesarean section after prolonged rupture of membranes (>4 hours) [5, 30, 31]. Of these 27 infants, (7.4%) died during the follow-up period. The cause of death was pneumonia in one case and possible pneumonia in the other. It is uncertain if either infant was exposed to HSV during birth, however, as the genital swabs were obtained thirteen and five days, respectively, prior to the delivery date. For the other 25 potentially exposed neonates, 13 were healthy at the postpartum visit, six additional neonates were doing well on follow-up phone call (range, 10–21 days after delivery), and six were lost to follow-up.

## 4. Discussion

Our study demonstrates that prior HSV-2 infection is frequent among pregnant women in South Africa, as is genital HSV shedding during labor, especially among HIV-positive women. Among HSV-2 seropositive women, the shedding frequency is similar to women in the USA during labor, where genital HSV was detected in 30.8% of HIV-positive and 9.5% of HIV-negative women [32, 33]. Our study suggests that the lack of published reports of neonatal herpes in South Africa is not due to infrequent exposure to HSV-2 during birth.

While we did not identify any cases of overt neonatal herpes, the study was not powered to estimate the frequency of neonatal herpes. The two infants born vaginally to women with HSV-2 reactivation at enrollment (five and thirteen days prior to delivery) were unlikely to be infected with HSV. While pneumonia can be a feature of disseminated HSV infection, this typically occurs at days 10–12 of life [2]. A systematic evaluation of acutely ill neonates, with diagnostic testing by HSV PCR or viral culture, would be required to accurately estimate the incidence of neonatal herpes in developing countries.

We collected genital specimens from women thought to be in early labor, but only half of women are known to have delivered within one day of genital swab collection. For the 119 HSV-2 seropositive women who delivered within one day of sampling, the HSV-2 shedding rate was 17%, the same as the larger cohort. Thus, it is clear that exposure to HSV-2 during birth occurs frequently in infants in South Africa. Our ability to determine which neonates were at greatest risk for neonatal herpes due to contact with HSV-2 during delivery was limited. In immunocompetent persons, with daily swab collection, subclinical shedding episodes last a median duration of only 2 days (IQR, 1.0–3.5) [16]. Studies with more frequent sampling (four times daily) in HIV-positive and immunocompetent persons have suggested that half of episodes may be <12 hours in duration [17, 34]. The low rate of HSV-2 detection among HSV-2 seropositive women with genital lesions in our study suggests that rapid clearance of HSV-2 by pregnant women may be common. To more accurately assess which neonates are at greatest risk for neonatal herpes, genital swabs for HSV PCR would need to be collected closer to the time of delivery in a larger cohort of women.

Although 41% of participants were HSV-2 seronegative at enrollment, we did not detect any episodes of HSV-2 acquisition in late pregnancy: no women seroconverted and HSV-2 shedding was detected in no HSV-2 seronegative women. In neighboring Zimbabwe, the incidence of HSV-2 seroconversion was 1.8% between 36 weeks of gestation and 6 weeks after delivery [35] and among HIV-positive women, 17.3% seroconverted between delivery and 6 weeks after delivery [36]. These rates are considerably higher than in the USA, where between the first prenatal visit and delivery (median interval of 6.5 months) the HSV seroconversion rate is 1.3%, with 1.0% developing HSV-2 antibodies [7]. Although we have complete data on genital HSV-2 shedding in HSV-2 seronegative women, the expected shedding rate with recent HSV-2 acquisition is less than 50% [37]; first episode infections in late pregnancy, therefore, may not have been captured by detection of genital HSV shedding alone. Our incomplete follow-up, however, limits the number of women evaluated for seroconversion. With an estimated HSV-2 incidence of 5–10% [14], only 1.3 to 2.5% of the study population would be expected to have acquired HSV-2 in the past three months. While no participants seroconverted, the 95% confidence interval (0–3.2%) includes the percentage of women expected to seroconvert if annual HSV-2 incidence was 10% and remained unchanged in late pregnancy.

## 5. Conclusions

Despite the limitations in detecting HSV-2 acquisition in late pregnancy, this study demonstrates a high rate of genital HSV-2 shedding during labor in HSV-2 seropositive women in South Africa. While the risk of neonatal transmission for women with HSV reactivation is thought to be <1% [4] due to protection by maternal neutralizing antibodies, 20–50% of neonatal herpes cases in the USA and Europe are associated with HSV-2 reactivation [6–8] due to the high prevalence of HSV-2 relative to the low incidence of HSV-2 in late pregnancy. If the absence of published reports of neonatal herpes from South Africa reflects decreased neonatal infection with HSV, as opposed to decreased identification and reporting, given the high genital shedding rate that we have found among HSV-2 seropositive women, there may be differences in the transmissibility of HSV-2 to the neonate due to virologic, host, or iatrogenic (e.g., decreased use of invasive fetal monitoring devices) factors. Studies investigating HSV-2 shedding patterns, genetic differences in HSV-2 strains, and immunologic differences between South African and USA mother-infant pairs may provide insights into the pathogenesis of neonatal HSV.

## Figures and Tables

**Table 1:** Baseline participant characteristics.

Characteristic	HIV-positive *n* = 132 (34.1%)	HIV-negative *n* = 255 (65.9%)	Total *n* = 390
Age, mean (range), yrs	29 (18–40)	26 (18–44)	27 (18–44)
Gravidity, *n* (%)			
Primigravid	23 (17.6)	112 (44.8)	135 (35.4)
Multigravid	108 (82.4)	138 (55.2)	246 (64.6)
EGA at enrollment, mean (range), weeks	39 (32–44)	39 (30–42)	39 (30–44)
HSV serostatus by Western blot, *n* (%)			
HSV-1+ only	16 (12.1)	144 (56.5)^a^	161 (41.3)
HSV-2+ only	5 (3.8)	2 (0.8)	7 (1.8)
Both HSV-1+ and HSV-2+	111 (84.1)	109 (42.8)	222 (56.9)
History of genital ulcers among HSV-2 seropositive, *n* (%)	16 (14.2)	6 (5.5)	22 (9.9)
Genital lesions present among HSV-2 seropositive, *n* (%)	3 (2.6)	4 (3.6)	7 (3.1)
CD4 count, median (range), and cells/*µ*L^b^	321 (18–1237)		
Antiretroviral therapy			
HAART^c,d^			
Any CD4 count	52/132 (39.4)		
CD4 ≤ 350 cells/*µ*L	46/70 (65.7)		
CD4 ≤ 200 cells/*µ*L	21/26 (80.8)		
Prophylaxis to prevent mother-to-child transmission	78 (59.1)		
Total receiving antiretroviral drugs^e^	131 (99.2)		

Abbreviations: EGA: estimated gestational age; HAART: highly active antiretroviral therapy (≥ three antiretroviral drugs). Missing data: HIV-status (*n* = 3), gravidity (*n* = 9), EGA (*n* = 4), history of genital ulcers among HSV-2 seropositive (*n* = 7), and CD4 count (*n* = 6). Percentages determined by excluding those with missing data from the denominator. ^a^Includes one participant with HSV-1 and atypical HSV-2 bands on Western blot at baseline for whom we did not obtain a postpartum Western blot. ^b^CD4 count obtained a median of 99 days prior to enrollment (IQR, 57–129 days). ^c^Denominator is the number of participants with CD4 count within range specified. ^d^In April 2010, one month prior to initiation of this study, South African National Department of Health guidelines were revised to recommend HAART initiation for all pregnant women with a CD4 count ≤350 cells/*µ*L [27]; previous guidelines recommended HAART initiation for a CD4 count ≤200 cells/*µ*L or WHO stage IV disease. ^e^Total receiving antiretroviral drugs does not equal those receiving HAART+ those receiving prophylaxis to prevent mother-to-child transmission as the only antiretrovirals recorded for one participant were zidovudine and lamivudine.

**Table:** (a)

Variable	Genital HSV-2 shedding among HSV-2 seropositive women^a,b^				HSV-2 seropositivity					
	Genital HSV-2 present, *n* = 39 *n* (%)	Genital HSV-2 not present, *n* = 188 *n* (%)	Unadjusted RR (95% CI)^c,d^	*P* value	HSV-2 seropositive, *n* = 229 *n* (%)	HSV-2 seronegative, *n* = 161 *n* (%)	Unadjusted RR (95% CI)^c^	*P* value	Adjusted RR (95% CI)^c,e^	*P* value
HIV status										
HIV-positive	26 (22.6)	89 (77.4)	1.91 (1.04, 3.53)	0.038	116 (87.9)	16 (12.1)	2.02 (1.73, 2.35)	<0.001	1.69 (1.45, 1.96)	<0.001
HIV-negative	13 (11.8)	97 (88.2)	1.00 (ref.)		111 (43.5)	144 (56.5)	1.00 (ref.)		1.00 (ref.)	
Age, per each 10 y increase			0.68 (0.41, 1.13)	0.14			1.62 (1.45, 1.82)	<0.001	1.28 (1.12, 1.46)	<0.001

Gravidity										
Primigravid	10 (24)	32 (76)	1.0 (ref.)		42 (31.1)	93 (68.9)	1.00 (ref.)		1.00 (ref.)	
Multigravid	28 (15.5)	153 (84.5)	0.65 (0.34, 1.23)	0.19	183 (74.4)	63 (25.6)	2.39 (1.84, 3.11)	<0.001	1.68 (1.26, 2.25)	<0.001

Abbreviations: RR: relative risk; HAART: highly active antiretroviral therapy (≥ three antiretroviral drugs). Missing data: genital HSV DNA (*n* = 2), HIV-status (*n* = 3), gravidity (*n* = 9), and CD4 count (*n* = 6). Percentages determined by excluding those with missing data from the denominator. ^a^HSV DNA was typed as HSV-2 in all cases. ^b^Genital HSV-2 DNA was not detected in any HSV-2 seronegative women. ^c^As determined by Poisson regression. ^d^Only HIV-status remained significantly associated with HSV-2 shedding after inclusion of maternal age and history of prior pregnancy in a multivariate model and backwards elimination of nonsignificant (*P* < 0.05) variables. ^e^Adjusted for HIV-status, age, and prior pregnancy.

**Table:** (b)

Variable	Genital HSV-2 shedding among HSV-2 seropositive women^a,b^				HSV-2 seropositivity			
	Genital HSV-2 present, *n* = 26 *n* (%)	Genital HSV-2 not present, *n* = 89 *n* (%)	Unadjusted RR (95% CI)^c^	*P* value	HSV-2 seropositive, *n* = 116 *n* (%)	HSV-2 seronegative, *n* = 16 *n* (%)	Unadjusted RR (95% CI)^c^	*P* value
CD4 count, cells/*µ*L								
<200	7 (28)	18 (72)	1.3 (0.5, 3.5)	0.62	25 (96)	1 (4)	1.1 (0.9, 1.3)	0.18
200–349	7 (20)	28 (80)	0.9 (0.3, 2.6)	0.87	36 (84)	7 (16)	1.0 (0.8, 1.2)	0.87
350–499	6 (23)	20 (77)	1.1 (0.4, 3.0)	0.91	26 (87)	4 (13)	1.0 (0.8, 1.3)	0.87
≥500	5 (22)	18 (78)	1.0 (ref.)		23 (85)	4 (15)	1.0 (ref.)	
Receiving HAART								
Yes	12 (26)	34 (74)	1.3 (0.7, 2.5)	0.47	47 (90)	5 (10)	1.0 (0.9, 1.2)	0.46
No	14 (20)	55 (80)	1.0 (ref.)		69 (86)	11 (14)	1.0 (ref.)	
HAART regimen containing tenofovir								
Yes	4 (22)	14 (78)	0.8 (0.3, 2.2)	0.64	19 (86)	3 (14)	0.9 (0.8, 1.1)	0.43
No	8 (29)	20 (71)	1.0 (ref.)		28 (93)	2 (7)	1.0 (ref.)	

Abbreviations: RR: relative risk; HAART: highly active antiretroviral therapy (≥ three antiretroviral drugs). Missing data: genital HSV DNA (*n* = 1), gravidity (*n* = 1), and CD4 count (*n* = 6). Percentages determined by excluding those with missing data from the denominator. ^a^HSV DNA was typed as HSV-2 in all cases. ^b^Genital HSV-2 DNA was not detected in any HSV-2 seronegative women. ^c^As determined by Poisson regression.

**Table 3:** Deaths known to have occurred among infants during the follow-up period, *n* = 10.

Maternal age (years)	Maternal comorbidities^a^	EGA (weeks)	Birth weight (g)	Delivery route	Maternal HSV-2 serostatus at delivery	Maternal genital HSV-2 (log_10_ copies/mL)	Days between delivery and swab collection	Age at death (days)	Cause of death
20	HIV (CD4 229) ARVs: 3TC, TDF, NVP	40	3150	Vaginal	Positive	2.28	13	35	Pneumonia
30	HIV (CD4 489) ARV: AZT	39	3130	Vaginal	Positive	2.56	5	36	Possible pneumonia
42	Gestational diabetes	37	2200	Vaginal	Positive	ND	50	Stillborn	
33	None	41	3971	Vaginal	Positive	ND	4	Stillborn	
22	None	37		Vaginal	Negative^b^	ND	0	Stillborn, twin survived	
23	None	42	3930	Vaginal	Positive	ND	1	0	Perinatal asphyxia
24	Epilepsy, treated with valproic acid	42	2325	Vaginal	Positive	ND	2	1	Respiratory failure
38	Pregnancy-induced hypertension	41	3230	Vaginal	Negative^b^	ND	2	4	Perinatal asphyxia
33	Hypertension	38	2220	Cesarean section	Positive	ND	4	8	Details not available
22	None	37	2780	Cesarean section	Negative^b^	ND	1	26	Congenital heart disease, pneumonia

Abbreviations: EGA: estimated gestational age; ARVs: antiretrovirals; 3TC: lamivudine; TDF: tenofovir; NVP: nevirapine; AZT: zidovudine. ND: not detected. ^a^All ten women had a nonreactive rapid plasma reagin (RPR) during the prenatal period. ^b^Postpartum HSV Western blot not available.
